# Octogenarians with EGFR-mutated non-small cell lung cancer treated by tyrosine-kinase inhibitor: a multicentric real-world study assessing tolerance and efficacy (OCTOMUT study)

**DOI:** 10.18632/oncotarget.23836

**Published:** 2018-01-02

**Authors:** Romain Corre, Radj Gervais, Florian Guisier, Louis Tassy, Florent Vinas, Régine Lamy, Gislaine Fraboulet, Laurent Greillier, Helene Doubre, Renaud Descourt, Christos Chouaid, Jean-Bernard Auliac

**Affiliations:** ^1^ Department of Pneumology, CHU Pontchaillou, Rennes, France; ^2^ Pneumo-Oncology Department, Centre Francois Baclesse, Caen, France; ^3^ Pneumology Department, CHU Hôpitaux de Rouen-Charles Nicolle, Rouen, France; ^4^ Oncology Department, Institut Paoli-Calmette, Marseille, France; ^5^ Pneumology Department, CH Intercommunal de Créteil, Créteil, France; ^6^ Oncology Department, CH Sud-Bretagne, Lorient, France; ^7^ Pneumology Department, Hôpital René-Dubos, Pontoise, France; ^8^ Pneumo-Oncology Department, Hôpital Sainte-Marguerite, Assistance Publique-Hôpitaux de Marseille, Marseille, France; ^9^ Pneumology Department, Hôpital Foch, Suresnes, France; ^10^ Cancerology Institute, CHU Brest, Brest, France; ^11^ Pneumology Department, CH François Quesnay, Mantes La Jolie, France; ^12^ UMR INSERM U1242-COSS, Rennes University, Rennes, France

**Keywords:** targeted therapies, tyrosine-kinase inhibitors, non-small cell lung cancer, EGFR, elderly, Gerotarget

## Abstract

**Objective:**

To assess efficacy and tolerance of EGFR tyrosine-kinase inhibitors (TKIs) for advanced EGFR-mutated non-small cell lung cancer (NSCLC) in octogenarians.

**Patients and methods:**

Patients aged 80 years or older with EGFR-mutated NSCLC treated by EGFR TKI between January 2011 and March 2015 whatever the line of treatment were retrospectively selected.

**Results:**

20 centers retrospectively included 114 patients (women, 77.2%; Caucasians, 98.3%; mean age, 83.9 years). A performance status of 0–1 or 2–3 at diagnosis was reported for 71.6% and 28.4% of patients, respectively. Overall, 95.6% of patients had adenocarcinomas and histological stage at diagnosis was stage IV for 79.8% of patients. EGFR mutations were identified mainly on exon 19 (46.5%) and exon 21 (40.4%). A geriatric assessment was performed in 35.1% of patients. TKI treatment was administered to 97.3% of patients as first or second line of treatment. Overall response rate and disease control rate were 63.3% (69/109) and 78.9% (86/109), respectively. Median progression-free survival was 11.9 months (95% confidence interval [CI], 8.6–14.7) and median overall survival was 20.9 months (95% CI, 14.3–27.1). After progression, 36/95 (37.9%) patients received a new line of chemotherapy. Main toxicities were cutaneous for 66.7% of patients (grade 3–4, 10%), diarrhea for 56.0% (grade 3–4, 15%; grade 5, 2%) and others for 25.7% (grade 3–4, 41%).

**Conclusions:**

Octogenarians with EGFR-mutated NSCLC treated by EGFR TKI had clinical outcomes and toxicity profile comparable to younger patients. Geriatric assessment appeared to be underused in this population.

## INTRODUCTION

Lung cancer is the most common cancer worldwide and is the leading cause of cancer death in Western countries [1]. Among the different forms of lung cancer, non-small cell lung cancer (NSCLC) is the most common (80-85%). However, NSCLC is often diagnosed when metastases are present or when the disease is at an advanced stage. In about half of the cases, NSCLC is diagnosed in patients >65 years and in 30-40% of the cases in patients >70 years [2]. With the growing of aging population, elderly patients with lung cancer will be more frequent [3].

According to the last guidelines from the European Society for Medical Oncology (ESMO), it is recommended to use carboplatin based-doublet treatment in fit elderly patients with NSCLC [4, 5]. In elderly patients who are less fit, single agent therapy (vinorelbine, gemcitabine or taxane) is an option [6-8]. Nevertheless, over 80 years, there are no firm recommendations because data on NSCLC treatment are scarce in this population of patients.

Erlotinib was the first EGFR tyrosine-kinase inhibitor (TKI) to be approved in advanced stage NSCLC after failure of first-line chemotherapy with platinum. The BR.21 study showed a benefit for progression-free survival (PFS) and overall survival (OS) with erlotinib after failure of first- or second-line therapy [9]. The drawback was a significant higher rate of grade 3-5 toxic effects in older patients (35% in patients ≥70 years vs. 18% in patients <70 years) [10]. In 2004, Lynch et al demonstrated that EGFR mutations were predictors of a response to EGFR TKI that, in addition, was often intense and fast [11]. Therefore, screening EGFR mutations offered the possibility to select patients who would respond to EGFR TKI. Many studies confirmed the superiority of EGFR TKIs for PFS in comparison with a platin based-doublet in patients with advanced NSCLC harboring an activating EGFR mutation in first-line setting [12-18]. On this basis, gefitinib, erlotinib and the more recent afatinib were approved for the first-line treatment of advanced NSCLC in patients harboring an activating EGFR mutation.

In addition, compare to chemotherapy [13-18] the safety profiles of the different EGFR TKIs were favorable. In this setting, few data are available in patients 80 years or more because clinical studies on EGFR TKI included few elderly patients. Thus, the upper limit of age for inclusion was 75 years in the studies of Maemondo *et al* [14] and Zhou *et al* [15] and 65 years for LUX-Lung 6 study [18]. Inoue *et al* demonstrated that elderly patients or patients with poor performance status with advanced NSCLC harboring EGFR mutation could benefit from EGFR TKI [19]. These results were confirmed in other Asian studies, but no data about Caucasian octogenarians were available [20, 21].

The aim of the OCTOMUT study was to improve knowledge on the efficacy and safety of EGFR TKIs in patients 80 years or more with advanced NSCLC harboring activating EGFR mutation.

## RESULTS

### Socio-demographic characteristics of patients

A total of 114 patients were selected by the 20 French participating centers. Three out four patients were women (77.2%) with a mean (SD) age of 83.9 (3.9) years and 98.3% were Caucasians (Table 1). They lived at home for 90.4% (including 45.6% with some help) and 9.6% lived in retirement home. Their performance status was 0-1 for 71.6% and 76.4% took a number of medications ≥3. A Charlson comorbidity index was available in only 14.0% of patients. A geriatric assessment was performed for only 35.1% of patients including Activities of Daily Living (ADL; *n* = 29), Instrumental Activities of Daily Living (IADLs; *n* = 27) and Mini Mental State (MMS; *n* = 25).

**Table 1:** Socio-demographic characteristics of octogenarian patients from OCTOMUT study

	N = 114
Age (years), mean (SD)	83.9 (3.9)
Women, n (%)	88 (77.2)
Body mass index (kg/m^2^)	
Mean (SD)	24.5 (3.9)
Classes, n (%)	
Leanness	2 (2.4)
Normal	46 (55.4)
Overweight	27 (32.5)
Obesity	8 (9.6)
*MD*	31
Caucasians, n (%)	112 (98.3)
Non-smokers, n (%)	87 (76.3)
Performance status, n (%)	
0–1	73 (71.6)
2–3	29 (28.4)
*MD*	12
Number of medications, n (%)	
0–2	25 (23.6)
3–5	41 (38.7)
≥6	40 (37.8)
*MD*	8
Way of life, n (%)	
Alone at home	94 (90.4)
Retirement home	10 (9.6)
*MD*	10
Charlson comorbidity index performed, n (%)	16 (14.0)
Geriatric assessment performed, n (%)	40 (35.1)

MD, missing data.

### Characteristics of NSCLC and EGFR TKI treatment

The median time between first symptoms and diagnosis was 55.0 days (Table 2). The histological diagnosis was adenocarcinoma in 95.6%. EGFR mutations were identified mainly on exon 19 (46.5%) and exon 21 (40.4%). Almost all patients (97.3%) received EGFR TKI as first (83.2%) or second line (14.1%) of treatment. The main EGFR TKIs prescribed were gefitinib (54.4%) and erlotinib (39.5%). The approved dose of EGFR TKI was administered to 87.2% (95/109) of patients.

**Table 2:** Characteristics of NSCLC in patients from OCTOMUT study

	N = 114
Median time (days) between first symptoms and diagnosis	55.0
Adenocarcinoma, n (%)	109 (95.6)
Histological stage at diagnosis, n (%)	
I–II	8 (7.0)
III	15 (13.2)
IV	91 (79.8)
Localization of metastases, n (%)	
Pleural cavity	43/91 (47.3)
Lung	29/91 (31.9)
Lymph nodes	18/91 (19.8)
EGFR mutation, n (%)	
Exon 19	53 (46.5)
Exon 21	46 (40.4)
Exon 18	9 (7.9)
Exon 20	6 (5.2)
Number of lines of treatment before EGFR TKI, n (%)	
0	94 (83.2)
1	16 (14.2)
≥2	3 (2.6)
*MD*	1
Type of EGFR TKI, n (%)	
Gefitinib	62 (54.4)
Erlotinib	45 (39.6)
Afatinib	2 (1.8)
Undefined	5 (4.2)

MD, missing data.

### Clinical efficacy outcomes after treatment with EGFR TKI

**Figure 1:**
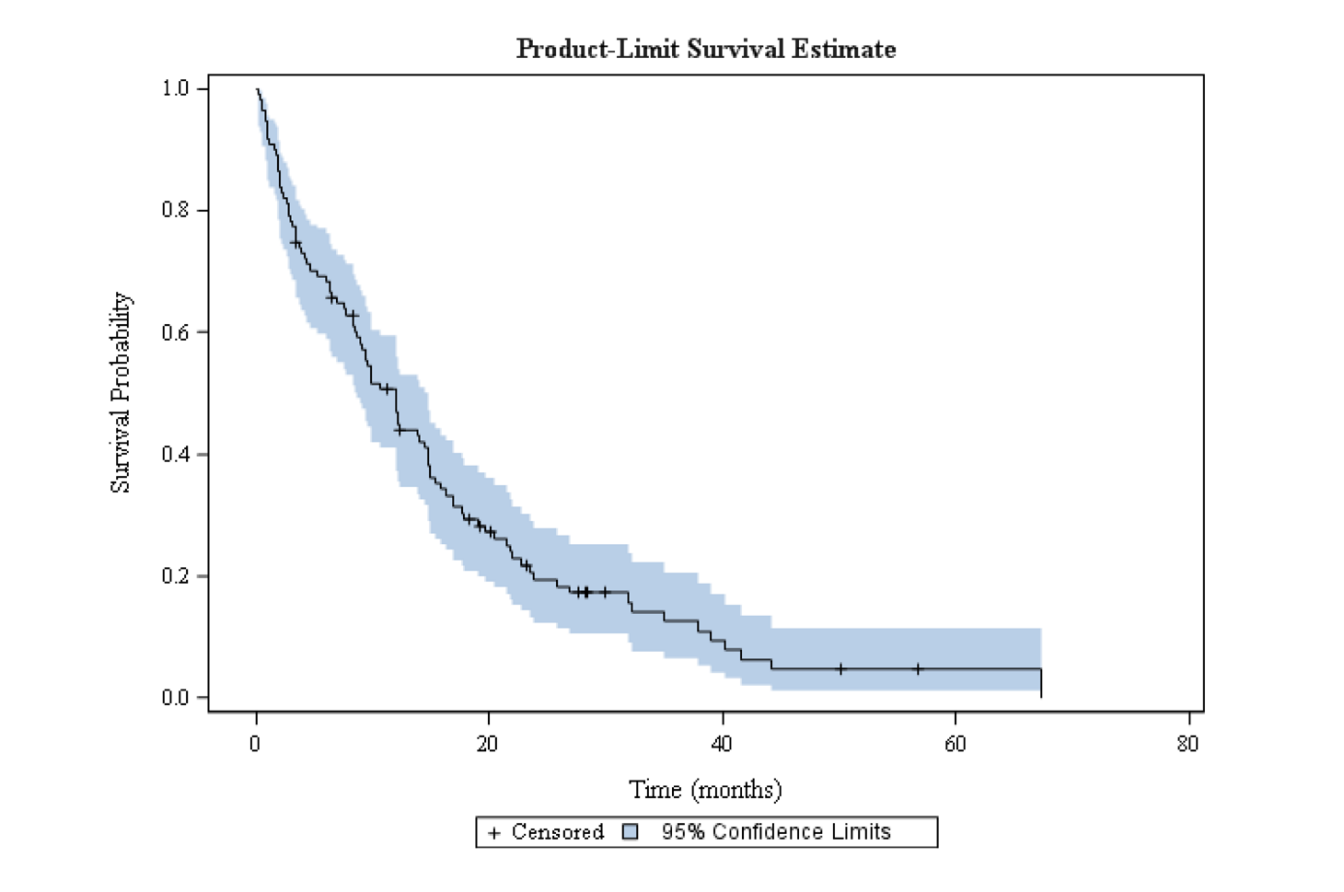
Progression-free survival in octogenarian patients with EGFR-mutated NSCLC treated with EGFR TKI

**Figure 2:**
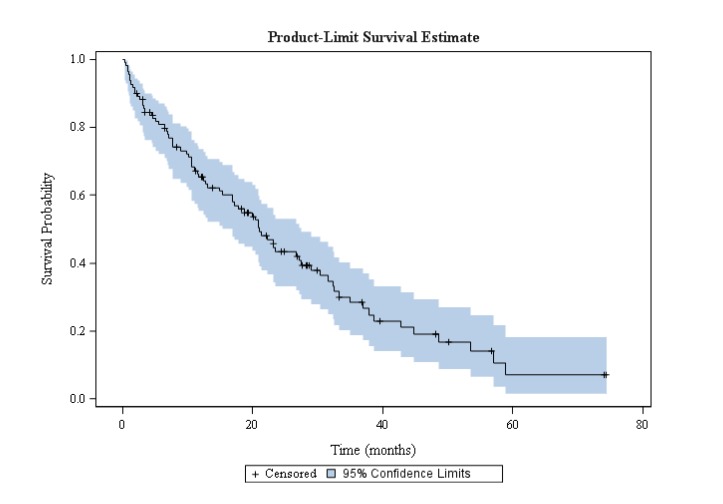
Overall survival in octogenarian patients with EGFR-mutated NSCLC treated with EGFR TKI

The overall response rate (ORR) was 63.3% (69/109) and the disease control rate was 78.9% (86/109) (Table 3). Median PFS was 11.9 months (95% confidence interval [CI], 8.6-14.7) and median OS was 20.9 months (95% CI, 14.3-27.1) (Figures 1-2). In 43.5% of patients who progressed according to RECIST criteria (34/78), EGFR TKI treatment was continued beyond progression for a median of 4.0 months (from 1 to 69 months); 36/95 (37.9%) of patients received chemotherapy after EGFR TKI discontinuation (platin-based doublet and monotherapy in 61% and 39% of cases, respectively) for a mean of 3 ± 2 cycles. Response and disease control rates of these chemotherapies were 30% and 39% respectively.

**Table 3:** Clinical efficacy outcomes in octogenarian patients after treatment with EGFR TKI (OCTOMUT study)

	N = 114
Best clinical response, n (%)	
Complete response (CR)	4 (3.7)
Partial response (PR)	65 (59.6)
Stable disease (SD)	17 (15.6)
Progressive disease (PD)	14 (12.8)
Not evaluated	9 (8.3)
*MD*	5
Overall response rate (ORR), n (%) ^a^	69 (63.3)
Disease control rate n (%) ^b^	86 (78.9)
Overall survival (months), median (95% CI)	20.9 (14.3–27.1)
Progression-free survival (months), median (95% CI)	11.9 (8.6–14.7)
Other line of treatment after progression, n (%)	36/95 (37.9)

MD, missing data

^a^ ORR = CR + PR

^b^ Disease control rate = CR + PR + SD.

### Toxicities of EGFR TKI

Main toxicities were cutaneous (66.7% of patients; grade 3-4, 10.3%), diarrhea (56%; grade 3-5, 17.0%) or others (25.6%; grade 3-4, 40.9%) (Table 4). We found no factor (ie: age, BMI or number of drugs…) significantly associated with a higher toxicity.

**Table 4:** Safety outcomes in the after treatment of octogenarian patient in the OCTOMUT study

	N = 114	Gefitinib N = 62	Erlotinib N = 45	Afatinib N = 7
Patients with report of toxicities	82/91 (90.1)	39/44 (88.6)	37/40 (92.5%)	6/7 (85.7%)
Cutaneo-mucous toxicity, n (%)	58/81 (71.6)	28/44 (63.7)	24/31 (77.4)	6/6 (100)
Grade 1	32/58 (55.2)	18/30 (60)	13/22 (59)	1/6 (16.7)
Grade 2	20/58 (34.5)	8/30 (26.7)	8/22 (36.3)	4/6 (66.6)
Grade 3	6/58 (10.3)	2/30 (6.7)	3/22 (13.7)	1/6 (16.6)
Digestive toxicity (diarrhea), n (%)	47/84 (56.0)	21/48 (43.7)	20/30 (66.7)	6/6 (100)
Grade 1	18/47 (38.3)	9/21 (42.9)	8/20 (40)	1/6 (16.7)
Grade 2	21/47 (44.7)	10/21 (47.6)	8/20 (40)	3/6 (50)
Grade 3	7/47 (14.9)	3/21(14.2)	2/20 (10)	2/6 (33.3)
Grade 5	1/47 (2.1)	0/25 (0)	0/16 (0)	1/6 (16.7)
Other toxicities, n (%)	19/74 (25.7)	8/39 (25.6)	6/30 (20)	5/5 (100)
Grade 1	4/22 (18.2)	2/8 (25)	1/6 (16.7)	1/5 (20)
Grade 2	9/22 (40.9)	4/8 (50)	3/6 (50)	2/5 (40)
Grade 3	8/22 (36.4)	4/8 (50)	3/6 (50)	1/5 (20)
Grade 4	1/22 (4.6)	0/8 (0)	1/6 (16.7)	0/5 (0)

## DISCUSSION

Although EGFR TKIs such as erlotinib, gefitinib or afatinib dramatically changed the history of metastatic NSCLC harboring EGFR mutations, not enough data are available on the efficacy of these targeted therapies in elderly patients and more specifically in Caucasian patients. Indeed, old age frequently prevents the realization of some diagnostic investigations and prescription of specific treatments because of fear of healthcare professionals or patients for excess toxicity and low efficiency. In clinical trials, difficulties in recruitment, management and follow-up lead to an under-representation of elderly cancer patients [23].

To our knowledge, the present retrospective multicenter study is the largest cohort dedicated to elderly Caucasian patients with NSCLC harboring activating EGFR mutations. This population was fragile: 45.3% of patients needed assistance at home, 28.4% had performance status 3-4 and their mean age was 83.9 years. Nevertheless, there was a clear benefit of EGFR TKIs in terms of survival. The ORR and the disease control rate were 63.3% and 78.9%, respectively. Median PFS and median OS were 11.9 months and 20.9 months, respectively. These results are in line with previous studies performed in Asian elderly patients and with a recent meta-analysis [19-21, 24].

Thus, Inoue *et al* reported that elderly patients or patients with poor performance status with advanced NSCLC harboring EGFR mutation could benefit from gefitinib treatment [19]. The ORR in these 30 patients was 66% and the disease control rate was 90%. The median PFS and median OS were 6.5 and 17.8 months, respectively. Some patients became eligible to a second-line chemotherapy treatment beyond disease progression. The authors concluded that examination of EGFR mutations as a biomarker was recommended in this patient population that was considered ineligible to chemotherapy because of their age or poor PS.

In another Asian prospective study, Maemondo *et al* reported efficacy results in 31 elderly patients with an age from 75 to 87 years with advanced NSCLC associated to activating EGFR mutations treated in first line by gefitinib [20]. The ORR was 74% and the disease control rate was 90%; the median PFS was 12.3 months. The authors concluded that considering the strong antitumor activity of gefitinib and its mild toxicity, first-line EGFR TKI might be preferable to standard chemotherapy for the elderly population.

The Asian study of Tateishi *et al* retrospectively analyzed the efficacy and safety of gefitinib in 55 patients from 75 to 94 years [21]. The ORR and disease control rate were 72.7% and 92.7%, respectively; the PFS and OS were 13.8 and 29.1 months, respectively.

The meta-analysis of Roviello *et al* reported the pooled results of five clinical trials with the use of EGFR TKI in EGFR-mutated NSCLC in first line [24]. Four phase III studies and one phase IIb study were included in the analysis for a total of 1381 patients [15, 18, 25-27]. Except the EURTAC study who included European patients and the LUX-Lung 7 studies who included both European and Asian patients, the other studies included exclusively Asian patients. Of interest, EGFR TKIs were more effective in prolonging PFS in elderly patients (≥65 years), with HR 0.39 (*p* = 0.008) compared with younger patients (< 65 years) with HR 0.48 (*p* = 0.04). In our study, 25.4% of patients continued EGFR TKI treatment after progression for a median of 4 months. These results were in line with studies [28, 29]suggesting under certain circumstances, that TKI treatment continuation after RECIST progression is an acceptable option in EGFR-mutated NSCLC patients.

Safety data’s in this octogenarian population were consistent with adverse events reported in phase III trials and in younger patients [13-18]. Cutaneo-mucous toxicity was reported in 66.7% of patients and digestive toxicity (diarrhea) in 56.0%. In the study of Maemondo *et al* rash was reported in 71.1% of patients treated with gefitinib and diarrhea in 34.2% [20]. In the study of Zhou *et al*, rash was reported in 73% of patients treated with erlotinib and diarrhea in 25% [15].

Before the arrival of the EGFR TKIs, an age >75 years was considered as a major drawback for the use of non-specific cytotoxic agents. Our study clearly shows that patients over 80 years can be treated with EGFR TKI with results that were satisfactory in term of efficacy and safety. Of note, comorbidities and geriatric assessment were insufficiently considered:only 14.0% of patients with a Charlson comorbidity index and 35.1% with a geriatric assessment. Even if, in France, it is recommended to perform a geriatric assessment for elderly patients with cancer, ≥75 years old and with a G8 questionnaire score ≤14/17. This was not always done most of the time by lack of geriatricians resource, and probably also because the treatment was an oral targeted therapy and not an intra-venous chemotherapy. Yet, our group showed in a large phase III randomized study that a geriatric assessment, performed by the pneumo-oncologists, improved the tolerance to chemotherapy in elderly patients treated for advanced lung cancer [30]. Additional studies are needed to assess the impact of geriatric assessment in patients receiving oral cancer treatment, more particularly EGFR TKIs and also to identify geriatric parameters associated with tolerance and/or survival.

About three out of four patients took a number of medications 3 suggesting potential drug interactions with EGFR TKIs. A careful search must be systematically performed in order to improve the efficacy and safety of these treatments [31].

This study has some limitations. The data were recorded retrospectively from patient medical files. These records were not designed for this analysis and the quality of data can be questioned, more particularly for adverse events that could have been underestimated. Patients were selected if they could give their consent (an exemption was obtained for dead patient). Therefore, we cannot exclude a bias with patients lost to follow-up. This possible bias is probably limited since patients over 80 years with EGFR-mutated NSCLC and treated by EGFR TKI were not numerous in each center and they benefited from a very regular follow-up. Another limitation is the absence of comparator group. Finally, considering the inclusion period, mechanisms of resistance to EGFR TKIs that led to progression were not explored as the current recommendations.

In conclusion, in this real-world analysis, octogenarians with EGFR-mutated advanced NSCLC treated by EGFR TKI had clinical outcomes and toxicity profile comparable to younger patients in previous studies. Therefore, elderly should not be a limiting factor for EGFR TKI treatment. Geriatric assessment appeared nevertheless to be underused in this population.

## MATERIALS AND METHODS

### Study design and patients

OCTOMUT was an observational multicentric retrospective study performed in French centers belonging to the French Group of Lung Cancer (GFPC). GFPC is a non-profit federation of university hospitals, general hospitals or healthcare centers that manage patients with thoracic cancers.

Main inclusions criteria’s were patients aged 80 and older with EGFR-mutated advanced NSCLC treated with EGFR TKI between January 1, 2011 and March 31, 2015 whatever the line of treatment. Patients were chronologically enrolled during the selection period. Data were retrospectively obtained from medical records.

The study was conducted in accordance with the Declaration of Helsinki and was approved by local independent Ethics Committee (Saint-Etienne University Hospital; IRBN 112016/CHUSTE). Written informed consent was obtained from each patient. For dead patients, an exemption of family consent was obtained from the National Commission for Data Protection and Liberties (CNIL).

### Study endpoints

The primary endpoint was OS and the main secondary endpoints were progression-free survival (PFS); best response according to RECIST 1.1 criteria; toxicity according to NCIC-CTC toxicity criteria (version 4.0) [22].

### Statistical analyses

It was planned to enroll 100 patients. In the previous Asian studies of Maemondo *et al* [20] and Tateishi *et al* [21], 31 and 55 patients were included. Moreover, an exploratory analysis performed over the period 2010-2012 in a limited number of GFPC centers retrieved 34 patients ≥80 years treated with EGFR TKI. Therefore, it was calculated that 30 GFPC centers would be able to enroll 100 patients. The analysis was essentially descriptive and no formal hypothesis was tested. Qualitative data were described by their frequency, percentage and 95% confidence interval (CI). Quantitative data were described by their mean and standard deviation or median and interquartile range. Analyses of OS and PFS were conducted using the Kaplan-Meier method. For OS, time-to-event was from the date of the histological diagnosis to the date of death. For PFS, time-to-event was from the first day of TKI treatment to the date of progression or death. Analyses were performed using SAS software version 9.2 (SAS Institute, Inc., Cary, North Carolina, US).

## Abbreviations

TKIs: tyrosine-kinase inhibitors
NSCLC: non-small cell lung cancer
GFPC: French Group of Lung Cancer
ESMO: European Society for Medical Oncology
PFS: progression-free survival
OS: overall survival
ADL: Activities of Daily Living
IADL: Instrumental Activities of Daily Living
MMS: Mini Mental State
